# The Influence of Orthopedic Journals in Knee Arthritis Treatment Research: Evaluation Using Relative Citation Ratio

**DOI:** 10.7759/cureus.23415

**Published:** 2022-03-23

**Authors:** Martinus Megalla, Zachary T Grace, Ali M Omari, Angeline Sanders, Nareena Imam, John D Koerner, Frank G Alberta, Gregg R Klein

**Affiliations:** 1 Orthopedics, Hackensack Meridian School of Medicine, Nutley, USA; 2 Orthopaedic Surgery, Rothman Orthopaedic Institute, Philadelphia, USA; 3 Office of Clinical Research Administration, Hackensack University Medical Center, Hackensack, USA; 4 Orthopaedic Surgery, Hackensack University Medical Center, Hackensack, USA

**Keywords:** treatments, orthopedics, arthritis, knee, bibliometrics

## Abstract

Introduction

The iCite database, developed by the National Institute of Health (NIH), utilizes a bibliometric known as the relative citation ratio (RCR) to gauge scholarly impact. The goal of this study was to use the RCR to evaluate the influence of orthopedic journals in regard to knee arthritis treatment literature, as no such studies exist to date.

Materials and methods

The 100 highest RCR-rated articles published between 2007 and 2017 were obtained in the following categories: physical therapy (PT), viscosupplementation (VS), nonsteroidal anti-inflammatory drugs (NSAIDs), corticosteroid injection (CSI), results of total knee arthroplasty (TKA), platelet-rich plasma (PRP), and meniscectomy (MS). Journals were categorized with respect to the following specialties: general orthopedics (GO), orthopedic subspecialty (OSS), nonsurgical musculoskeletal (NSMSK), general medicine (GM), and basic science/nonclinical (BS/NC).

Results

Across the seven domains, GO journals held the highest median RCR, while OSS ranked fourth (RCR, 6.60 versus 3.95; p=0.0027). GO journals were considered the most influential specialty in CSI (RCR, 2.99), while OSS journals held the highest median RCR in PRP (RCR, 4.10). OSS and GO journals ranked third (RCR, 4.79) and fourth (RCR, 4.21), respectively, in NSAIDs, lagging behind NSMSK and GM journals.

Conclusions

Bibliometric tools, such as the RCR, can inform the orthopedic field of current and future research trends and help guide further research efforts. Currently, publications in GO journals hold a strong influence in CSI but less so in PT and NSAIDs. The use of bibliometrics allows the identification of highly influential non-orthopedic articles and journals to read while identifying influential non-orthopedic researchers to promote interdisciplinary collaboration.

## Introduction

Journal impact factor (JIF) is often used as a measure of a journal’s influence. JIF is calculated by dividing the number of citations in a given year by the number of “citable” articles published by that journal in the previous two years [1]. Although JIF is certainly a useful metric and often considered when authors determine where to submit their research, it does have several limitations. JIF is a mathematical average and therefore disproportionately weighs a small number of highly cited papers (e.g., review articles) and may falsely imply that papers with fewer citations are relatively unimportant [2]. In addition, although JIF is a measure of journal impact, it is frequently used to determine the impact of an individual author’s research and may influence decisions such as hiring or promotions [1,2].

In recognition of the limitations of JIF and other citation metrics, the National Institute of Health (NIH) developed a metric known as the relative citation ratio (RCR). The RCR is calculated as the article citation rate divided by the expected citation rate, which is based on each article’s co-citation network [3]. The RCR metric is article- and field-independent and serves as an alternative measure of an article’s influence. RCR may also be useful in measuring the impact of journals by assessing which journals contain the most influential articles. Several studies have used RCR to assess the influence of authors in their respective research fields, including vascular surgery [4], urology [5], and psychiatry [6].

There is significant overlap in regard to the diagnosis and management of many musculoskeletal conditions between orthopedic surgeons and other specialties. In addition, researchers across multiple disciplines publish work related to these issues. To date, there have been no studies utilizing RCR to evaluate the influence of orthopedic journals relative to other journals. The goal of this study is to use the RCR to evaluate the influence of orthopedic journals in knee literature, with a focus on the treatment of knee arthritis.

## Materials and methods

This study was exempt from institutional review board (IRB) approval as all data were obtained from publicly available databases and no patient information was collected. Using the iCite database, the top 100 highest RCR-rated articles published between 2007 and 2017 were obtained using research terms relevant to knee arthritis treatments in the literature: physical therapy (PT), viscosupplementation (VS), nonsteroidal anti-inflammatory (NSAIDs), corticosteroid injection (CSI), results of total knee arthroplasty (TKA), platelet-rich plasma (PRP), and meniscectomy (MS). These terms were chosen as they represent some of the most common treatment modalities for knee arthritis. Journals were categorized with respect to the following specialties based on the general scope of the journal: general orthopedics (GO), orthopedic subspecialty (OSS), nonsurgical musculoskeletal (NSMSK), general medicine (GM), and basic science/nonclinical (BS/NC). Examples of journals included in each of these specialty categories can be found in Table 1. Using the InCite™ Journal Citation Report® (Thomson Reuters Corporation, Toronto, Canada), we obtained the most recent five-year journal impact factor (JIF) for each journal.

**Table 1:** Examples of Journals Included in Each Specialty Category

General Orthopedics	Orthopedic Subspecialty	Nonsurgical Musculoskeletal	General Medicine	Basic Science/Nonclinical
International Orthopaedics	American Journal of Sports Medicine	Annals of the Rheumatic Diseases	New England Journal of Medicine	Biomaterials
Journal of the American Academy of Orthopaedic Surgeons	Journal of Knee Surgery	Osteoarthritis and Cartilage	Annals of Internal Medicine	Nutrition Journal
The Journal of Bone and Joint Surgery	Arthroscopy	Physical Therapy	Journal of the American Medical Association	International Journal of Molecular Sciences
Clinical Orthopaedics and Related Research	The Journal of Arthroplasty	Pain	British Medical Journal	International Journal of Pharmaceutics

After conducting the search for each topic, articles were evaluated for relevance, and those deemed not relevant were excluded by the senior orthopedic surgeons (FA and GK) with two surgeon consensus. The remaining 100 articles in each category with the highest RCR were used. Additional exclusion criteria included articles not available in English and nonhuman studies. Furthermore, journals that were either not included in the InCite database or did not have a five-year JIF were excluded from the analysis.

Statistics

All data were compiled into a master database using Microsoft Excel (Microsoft Corporation, Redmond, WA, USA). The Shapiro-Wilk test was used to test the normality of RCR and JIF. Due to the skewness of the data, medians were calculated for each journal specialty both overall and within each research term. RCR and JIF were compared among the journal specialties using the Kruskal-Wallis test. To investigate pairwise comparisons, Dunn’s test with Holm’s adjustment was performed. The Spearman correlation coefficient and a 95% confidence interval (CI) based on Fisher’s z transformation were calculated to evaluate the relationship between RCR and JIF for all 700 articles. All analyses were performed in R version 3.6.2. The “FSA” package [7] was used for Dunn’s multiple comparisons, and the “DescTools” package [8] was used for the Spearman correlation and 95% CI. A p-value of less than 0.05 indicated statistical significance.

## Results

**Figure 1:**
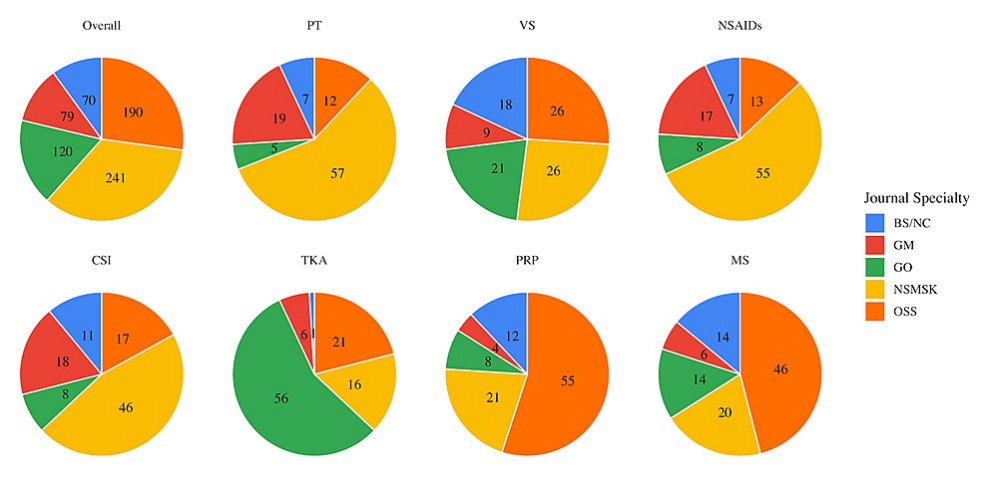
iCite Database Article Representation by Journal Specialty

Journal categorization of the 700 selected articles (100 for each of the seven search topics) yielded the following results: 241 NSMSK (34.4%), 190 OSS (27.1%), 120 GO (17.1%), 79 GM (11.3%), and 70 BS/NC (10.0%). For specific research terms, the NSMSK journal subcategory had the highest number of articles in three domains: PT (57/100), NSAIDs (55/100), and CSI (46/100). GO was found to be the highest contributing specialty journal for TKA (56/100), while OSS dominated the PRP (55/100) and MS (46/100) terms (Figure 1).

**Figure 2:**
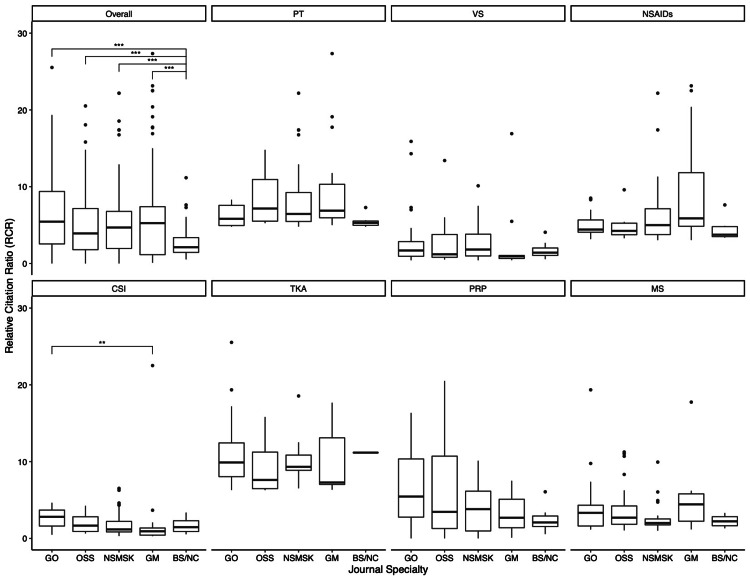
RCR Values by Journal Specialty and Knee Arthritis Research Term Abbreviations: PT, physical therapy; VS, viscosupplementation; NSAIDs, nonsteroidal anti-inflammatory drugs; CSI, corticosteroid injection; TKA, results of total knee arthroplasty; PRP, platelet-rich plasma; MS, meniscectomy; GO, general orthopedics; OSS, orthopedic subspecialty; NSMSK, nonsurgical musculoskeletal; GM, general medicine; BS/NC, basic science/nonclinical *p<0.05 **p<0.01 ***p<0.001

Across the seven selected knee arthritis research terms, RCR significantly differed with respect to journal category (p<0.0001) (Table 2). GO journals had the highest median RCR, while OSS journals had the fourth highest (RCR, 6.60 versus 3.95; p=0.0027). The median RCR for articles published in BS/NC journals was only 2.12, which was substantially lower than all other journal categories (all, p<0.05) (Figure 2).

In relation to the four other specialty journal classifications, GO was considered the most influential in the CSI (RCR, 2.99) research term. When only compared to the OSS category, GO had greater median RCRs in four out of the seven research terms: VS (RCR, 1.71 versus 1.23; p=0.8397), CSI (RCR, 2.99 versus 2.00; p=1.000), TKA (RCR, 9.89 versus 7.61; p=0.6885), and MS (RCR, 3.12 versus 2.78; p=1.000). Within the CSI term, the median RCR was substantially larger for articles published in GO and OSS journals compared to those published in GM journals (RCR, 2.99 versus 0.83; p=0.0295; and RCR, 2.00 versus 0.83; p=0.0419, respectively) (Table 2).

**Table 2:** Median RCR and JIF Values Among Five Journal Specialty Categories Abbreviations: RCR, relative citation ratio; JIF, journal impact factor; GO, general orthopedics; OSS, orthopedic subspecialty; NSMSK, nonsurgical musculoskeletal; GM, general medicine; BS/NC, basic science/nonclinical; PT, physical therapy; VS, viscosupplementation; NSAIDs, nonsteroidal anti-inflammatory drugs; CSI, steroid injection; TKA, results of total knee arthroplasty; PRP, platelet-rich plasma; MS, meniscectomy ^a^Kruskal-Wallis p-value reported *p<0.05

Topic	RCR						JIF					
	GO	OSS	NSMSK	GM	BS/NC	p-value^a^	GO	OSS	NSMSK	GM	BS/NC	p-value^a^
Overall	6.60	3.95	4.73	5.20	2.12	<0.0001*	4.41	3.68	5.35	7.97	3.14	<0.0001*
PT	6.25	6.47	6.43	6.97	5.29	0.0870	4.70	4.12	5.71	7.97	2.73	<0.0001*
VS	1.71	1.23	1.82	0.93	1.42	0.4716	2.98	3.42	3.82	3.11	2.90	0.4688
NSAIDs	4.21	4.79	5.00	5.88	3.75	0.1949	4.12	3.68	5.71	7.97	4.41	<0.0001*
CSI	2.99	2.00	1.17	0.83	1.49	0.0152*	3.10	3.16	3.49	2.37	4.44	0.0929
TKA	9.89	7.61	9.32	7.28	11.17	0.3997	4.70	3.16	5.71	28.03	3.23	<0.0001*
PRP	2.95	4.10	3.81	2.12	2.19	0.2503	2.11	4.59	2.19	3.50	3.14	0.0090*
MS	3.12	2.78	2.01	4.44	2.23	0.2988	4.12	4.59	5.71	15.96	2.89	0.0002*

**Figure 3:**
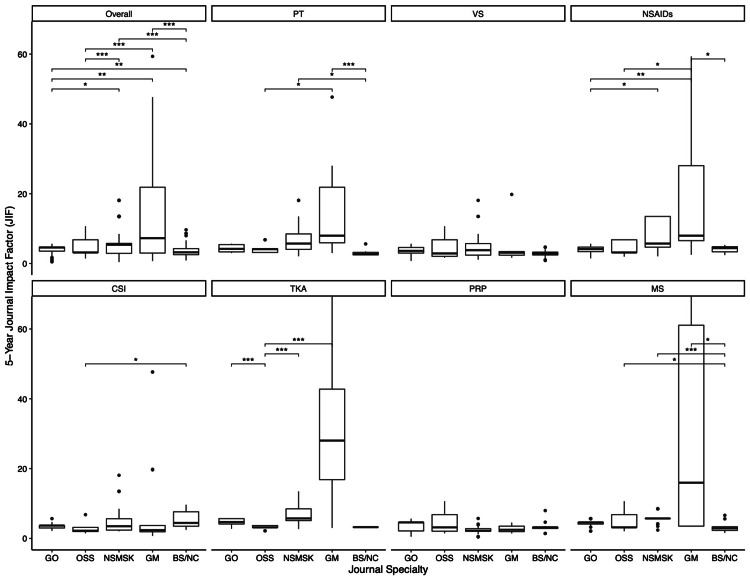
JIF Values by Journal Specialty and Knee Arthritis Research Term Abbreviations: RCR, relative citation ratio; JIF, journal impact factor; GO, general orthopedics; OSS, orthopedic subspecialty; NSMSK, nonsurgical musculoskeletal; GM, general medicine; BS/NC, basic science/nonclinical; PT, physical therapy; VS, viscosupplementation; NSAIDs, nonsteroidal anti-inflammatory drugs; CSI, steroid injection; TKA, results of total knee arthroplasty; PRP, platelet-rich plasma; MS, meniscectomy *p<0.05 **p<0.01 ***p<0.001

Across the seven knee search terms, five-year JIF differed significantly among the journal categories (p<0.0001) (Figure 3). GO journals held a higher median JIF compared to OSS journals (JIF, 4.41 versus 3.68; p=0.3877), although they were the third and fourth highest rated out of the five subcategories, respectively. Similar to RCR, BS/NC journals had the lowest median JIF at 3.14, which was significantly lower than GO (p=0.0112), OSS (p=0.0306), NSMSK (p<0.0001), and GM (p<0.0001) journals. Overall, GM had the greatest median JIF and was substantially larger than that of GO journals (JIF, 7.97 versus 4.41; p=0.0032) and OSS journals (JIF, 7.97 versus 3.68; p<0.0001). NSMSK journals also had higher median JIF compared to GO (JIF, 5.35 versus 4.41; p=0.0176) and OSS (JIF, 5.35 versus 3.68; p=0.0001) (Table 2).

Contrary to RCR, GO journals were not considered the most influential specialty group in any of the seven research terms with respect to JIF. When compared to OSS counterparts, GO journals carried a higher JIF in only three terms: PT (JIF, 4.70 versus 4.12; p=0.8669), NSAIDs (JIF, 4.12 versus 3.68; p=0.9923), and TKA (JIF, 4.70 versus 3.16; p<0.0001). The median JIF of GM journals was higher relative to that of OSS journals in the following research terms: PT (JIF, 7.97 versus 4.12; p=0.0048), NSAIDs (JIF, 7.97 versus 3.68; p=0.0018), and TKA (JIF, 28.03 versus 3.16; p<0.0001) (Table 2). Other comparisons are displayed in Figure 3.

**Figure 4:**
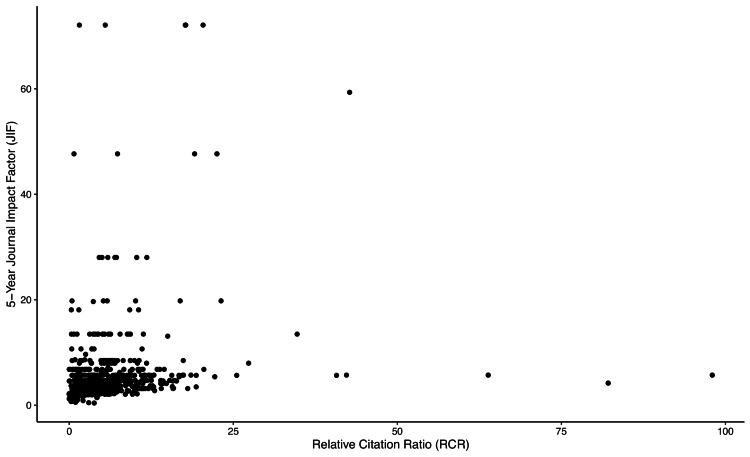
Scatterplot of RCR and Five-Year JIF Values for Orthopedic-Related Articles

To visually examine the correlation between RCR and JIF of all 700 orthopedic articles, a scatterplot was created (Figure 4). A weak to moderate relationship between RCR and JIF for the 700 orthopedic-related articles was identified (rs=0.38; 95% CI=0.318, 0.444; p<0.0001).

## Discussion

Our results highlight the influence of orthopedic journals in the literature with regard to various commonly used treatment modalities for knee arthritis. The relative strengths of GO journals reside primarily in the CSI and TKA domains when utilizing the RCR bibliometric. GO journals did not achieve the highest median JIF value in any of the seven research domains. For TKA, GO journals were behind BS/NC in having the highest median RCR value. However, it should be noted that there was only one article within TKA identified from a BS/NC journal, which may impact the ability to draw conclusions. Furthermore, GO journals were found to contribute the highest number of articles to the TKA category compared to the other specialty classifications. It is important to note that the relative strengths mentioned are in reference to the specific research terms chosen for this study and not necessarily representative of the global strengths of these journals.

Surprisingly, we found that neither of the orthopedic surgery journal categories were most influential in articles pertaining to arthroscopic meniscectomy (MS), a surgical procedure. For MS, GM journals held the highest influence in both RCR and JIF. However, this may be skewed due to small sample size and an outlier as only six articles from GM journals were included in this category as compared to 27 GO articles and 33 OSS articles. Nonetheless, data from the 2019 AAMC Physician Specialty Data Report indicates that there are 65,002 internal medicine, 16,768 orthopedic, and 3,687 rheumatology MD physicians currently practicing in the United States [9]. This difference in representation may help support the hypothesis that articles published in GM journals may reach a larger audience, which in turn may support higher citation rates and thus higher overall impact factor in relation to orthopedic-associated journals. As a result, it may be beneficial for orthopedic researchers to submit their high-quality work to journals with a broader audience.

These results also help recognize the contribution of non-orthopedic specialties to musculoskeletal research. There is significant overlap in the care of musculoskeletal conditions. As a result, there will be a wide array of non-orthopedic physicians who treat and ultimately add to the literature. Identifying non-orthopedic contributions to musculoskeletal research and, in particular, the treatment of knee arthritis can promote collaboration and sharing of knowledge between providers, which has been shown to improve patient outcomes [10]. Furthermore, orthopedic readers should also be encouraged to broaden their scope to general medicine, rheumatology, and other journals to enhance their educational experience. Conversely, general medicine and rheumatology readers may benefit from reading content in orthopedic journals.

The implementation of article- and journal-specific bibliometrics in the current study may also help inform future researchers of the most appropriate journals to seek publication in. For example, guidelines for the nonsurgical management of knee osteoarthritis by McAlindon et al. demonstrated the highest RCR value (97.99) out of the 700 total articles that were queried [11]. However, the peer-reviewed, publishing journal for the aforementioned article, Osteoarthritis and Cartilage, has a five-year JIF of 5.71 (in relation to the New England Journal of Medicine’s JIF of 72.1). While it may be true that the prestige of a journal may be tied to the publication of high-value articles, there is a considerable degree of randomness often overlooked during editorial selection that may cause important literature to remain unseen [12]. Fundamentally, evaluating papers based solely on which journals have published them is more likely to generate incorrect evaluations of those articles [12].

Consideration should also be directed to the vast amount of general medicine- and musculoskeletal-related research in comparison to the field of orthopedics. From a 10-year period from 2000 to 2010, the United States dominated output within orthopedic research, with a 10.2% annual increase in the number of publications [13]. While these expansive trends in scholarship are not limited to orthopedics, it is valid to add context to explain why fields such as general and nonsurgical musculoskeletal medicine were found to be more influential in categories such as PT, VS, NSAIDs, and MS. In terms of this study, both of the aforementioned groups were placed in larger, multispecialty categories. NSMSK included articles associated with rheumatology, arthritis, and nonoperative sports medicine, while GM included articles from hematology, family medicine, and pediatric journals. In essence, the orthopedic results demonstrated through this study may be subject to “crowding out” from other notable, highly achieving specialties that are playing a role in advancing knee arthritis research.

This study has several limitations. The arbitrary assignment of articles into journal specialty groups may create selection bias due to its subjective nature. Once assigned, articles were verified by the senior authors to limit this potential. Furthermore, employing a bibliometric such as the RCR to observe recent trends disadvantaged newer articles that may accrue citations at a slower rate in comparison to peer articles published at an earlier date [14]. We felt that using a 10-year period with a cutoff of 2017 would allow a sufficient citation period for more recent articles. There is also the possibility that the same articles appeared in multiple iCite database queries. Additionally, articles were not limited to one research term due to potential similarities between domains while attempting to uphold the natural algorithm of the iCite database.

From a statistical perspective, one limitation of this study is that some sample sizes within the knee arthritis research terms were small, directly impacting the statistical power of the tests performed. While we saw differences in the RCR and JIF magnitude between groups, the p-values were not always statistically significant. We reported all results from our analysis and indicated those differences that were statistically significant. In addition, we used the Holm method to adjust for multiple comparisons as it is uniformly more powerful than the commonly used Bonferroni method [15].

## Conclusions

Bibliometric tools such as the RCR can inform the orthopedic field of current and future research trends. Relative to other specialty journals in the area of treatment for knee arthritis, the current strengths of general orthopedic journals are focused on the corticosteroid injection domain. The use of the RCR prior to submission of manuscripts may help guide authors in their selection of journals to submit to in addition to providing evidence to guide resource allocation. Bibliometric analysis suggests that orthopedic surgeons would benefit from expanding their reading to include other highly contributing general medicine and nonsurgical musculoskeletal journals with regard to the study of treatment of knee arthritis.

## References

[1] Garfield E (2006). The history and meaning of the journal impact factor. JAMA.

[2] (2016). Time to remodel the journal impact factor. Nature.

[3] Hutchins BI, Yuan X, Anderson JM, Santangelo GM (2016). Relative citation ratio (RCR): a new metric that uses citation rates to measure influence at the article level. PLoS Biol.

[4] Davis FM, Obi AT, Gallagher KA, Henke PK (2020). Accessing the academic influence of vascular surgeons within the National Institutes of Health iCite database. J Vasc Surg.

[5] An JY, Baiocco JA, Rais-Bahrami S (2018). Trends in the authorship of peer reviewed publications in the urology literature. Urol Pract.

[6] Mohandoss AA, Thavarajah R (2016). Contribution of Indian psychiatrists to PubMed listed mental health literature during 1995-2013: an exploratory study. Indian J Psychol Med.

[7] Ogle DH PW, A. Dinno: FSA (2022). Fisheries Stock Assessment (FSA). https://github.com/droglenc/FSA.

[8] (2020). Tools for descriptive statistics and exploratory data analysis. https://andrisignorell.github.io/DescTools/.

[9] (2021). Active physicians with a U.S. doctor of medicine (U.S. MD) degree by specialty 2019. https://www.aamc.org/data-reports/workforce/interactive-data/active-physicians-us-doctor-medicine-us-md-degree-specialty-2019..

[10] Epstein NE (2014). Multidisciplinary in-hospital teams improve patient outcomes: a review. Surg Neurol Int.

[11] McAlindon TE, Bannuru RR, Sullivan MC (2014). OARSI guidelines for the non-surgical management of knee osteoarthritis. Osteoarthritis Cartilage.

[12] Starbuck WH (2005). How much better are the most-prestigious journals? The statistics of academic publication. Organ Sci.

[13] Lee KM, Ryu MS, Chung CY (2011). Characteristics and trends of orthopedic publications between 2000 and 2009. Clin Orthop Surg.

[14] Surkis A, Spore S (2018). The relative citation ratio: what is it and why should medical librarians care?. J Med Libr Assoc.

[15] Holm S (1979). A simple sequentially rejective multiple test procedure. Scand J Statist.

